# Silent Effects of High Salt: Risks Beyond Hypertension and Body’s Adaptation to High Salt

**DOI:** 10.3390/biomedicines13030746

**Published:** 2025-03-18

**Authors:** Raisa Nazir Ahmed Kazi

**Affiliations:** Department Respiratory Therapy, College of Applied Medical Sciences, King Faisal University, Al-Ahsa 37912, Saudi Arabia; rnahmed@kfu.edu.sa

**Keywords:** salt, hypertension, sodium intake, blood pressure, renal function, endothelial dysfunction, sympathetic overactivity

## Abstract

Hypertension is a major contributor to heart disease, renal failure, and stroke. High salt is one of the significant risk factors associated with the onset and persistence of hypertension. Experimental and observational studies have confirmed cardiovascular and non-cardiovascular detrimental effects associated with chronic intake of high salt. Because of convenience and present urban lifestyles, consumption of fast food has led to daily salt intake above the recommended level by the World Health Organization. This study provides an understanding of the body regulatory mechanisms that maintain sodium homeostasis under conditions of high salt intake, without health consequences, and how these mechanisms adapt to chronic high salt load, leading to adverse cardiovascular, renal, and non-cardiovascular outcomes. Recent research has identified several mechanisms through which high sodium intake contributes to hypertension. Of them, heightened renin–angiotensin–aldosterone and sympathetic activity associated with impaired pressure diuresis and natriuresis and decreased renal excretory response are reported. Additionally, there is the possibility of endothelial and nitric oxide dysfunction leading to vascular remodeling. These changes raise cardiac output and peripheral vascular resistance. Knowing how these collective mechanisms adapt to chronic intakes of high salt helps develop effective therapeutic policies to fight salt-induced hypertension.

## 1. Introduction

High dietary sodium intake has proven to be a significant modifiable lifestyle factor in the development of hypertension. A high salt diet not only increases blood pressure but also has a blood-pressure-independent effect on the heart, kidneys, and blood vessels. The World Health Organization (WHO) recommends limiting salt consumption to less than 5 g per day to improve BP control [1]. However, the precise mechanisms of how increased salt intake leads to the development of salt-dependent hypertension are not fully understood. Even though salt performs several essential functions in the body, including maintaining fluid balance (osmoregulation and hydration), supporting nerve function (action potential and neurotransmission), facilitating muscle function (muscle contraction), regulating blood pressure, and aiding nutrient transport in the digestive system (amino acid and glucose transport), excessive salt consumption can have detrimental effects. Most populations consume too much sodium, with the global mean intake for adults being 4310 mg/day (equivalent to 10.78 g/day of salt) [1,2,3]. This intake is more than double the WHO recommendation for adults, which is less than 2000 mg/day of sodium (equivalent to less than 5 g/day of salt). The primary health effect associated with high sodium diets is elevated blood pressure, which increases the risk of cardiovascular diseases and kidney disease. It is estimated that 1.89 million deaths each year are linked to excessive sodium consumption. Reducing sodium intake is one of the most cost-effective measures to improve health and reduce the burden of non-communicable diseases [4,5]. Foods with high sodium content include processed canned food, junk food, and use of table salt in excess at home. Quicker action is needed to decrease the consumption of high salt intake. In addition, increasing low-salt food and increasing consumption of vegetables and fruits is also the need of the hour. Salt restriction is considered a cost-effective measure to decrease high-salt-related hypertensive and cardiovascular and renal-disease-induced mortality and morbidity. The WHO is actively raising awareness about the importance of salt restriction, with a goal to reduce global sodium intake by 30% by the year 2025 [6]. For most of human evolution, salt consumption was less than 0.25 g per day [7,8]. High salt intake raises the risk of stroke, left ventricular hypertrophy, arterial stiffness, and kidney disease. Reducing salt intake by about 1.75 g of sodium per day (4.4 g of sodium chloride/day) has been shown to reduce systolic/diastolic blood pressure by an average of 4.2/2.1 mmHg, with a more pronounced effect (5.4/2.8 mmHg reduction) in hypertensive patients and considered a cost-effective method to lower high blood pressure and reduce cardiovascular complications. Therefore, reducing salt consumption should be a public health priority, requiring collaborative efforts between governments, food manufacturers, and the general population [6,7,8]. The objective of this study is to understand the normal and abnormal responses of the body to chronic salt intake and its underlying mechanisms. The comprehensive search used relevant MeSH terms, including sodium chloride (high salt), hypertension, renal insufficiency, cardiovascular diseases, endothelial dysfunction, blood pressure regulation, natriuresis, sympathetic nervous system, and vascular remodeling. Peer-reviewed studies in English (1986–2024) were sourced from PubMed, Scopus, and Google Scholar, ensuring credibility in cardiovascular, renal, and nutrition research. Under normal conditions, the body’s regulatory mechanisms maintain sodium homeostasis and hence prevent adverse cardiovascular and renal consequences. These mechanisms involve various neural and hormonal pathways to maintain blood pressure within a healthy range. However, when salt intake is excessively high, sodium starts to negatively impact cardiovascular function, primarily by raising blood pressure. Chronic excessive salt consumption increases blood volume and leads to sustained high blood pressure. As a result, the body’s regulatory mechanisms adapt to these elevated pressure levels, which over time can be harmful to both the cardiovascular system and kidneys [9,10]. Before going into the adverse effects of chronic high salt consumption on the body, it is essential to acknowledge the crucial role salt plays in our body and its importance. Without adequate salt, many of our physiological functions would be impaired.

**Figure 1:**
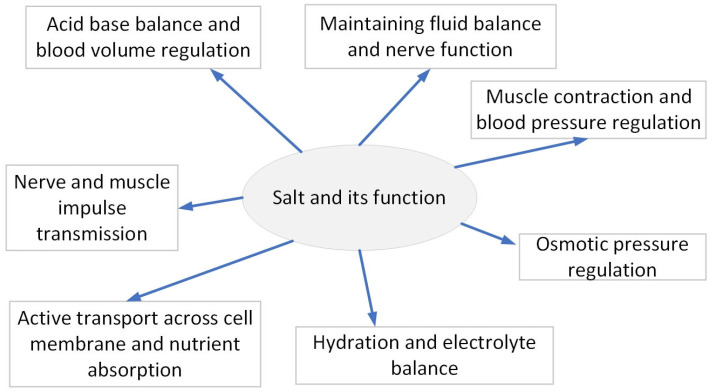
Role of salt in body.

Role of sodium in our body: The role of sodium in our body is crucial, as it regulates essential bodily functions (see Figure 1). However, it is important to consume sodium within the recommended limits. Excessive salt intake, particularly over a prolonged period, can have detrimental effects on health, with significant impacts on cardiovascular function.

## 2. The Essential Role of Salt in the Body (Figure 1)

Sodium is essential for maintaining various physiological functions within the body. It plays a key role in acid-base balance and blood volume regulation, where it helps buffer blood pH and manages blood volume, thereby influencing blood pressure. Sodium is crucial for fluid balance, as it regulates the distribution of fluids inside and outside cells, supporting hydration. Sodium is also involved in regulating osmotic pressure, ensuring that fluid concentrations across cell membranes remain balanced and preventing excessive cell swelling (endosmosis) or shrinking (exosmosis). For maintaining hydration and electrolyte balance, sodium chloride is essential for the equilibrium of electrolytes. Sodium plays a critical role in active transport across cell membranes and nutrient absorption. The sodium–potassium pump utilizes sodium to help transport nutrients like glucose into cells. Additionally, sodium and potassium ions work together to facilitate muscle contractions. The movement of sodium across cell membranes generates electrical charges (membrane potential), which are necessary for transmitting nerve and muscle impulses, promoting coordination and proper nervous system function [10]. Recent research has highlighted additional functions of salt that extend beyond the basics listed. Sodium has an important part to play in cognitive function, as the body requires an appropriate level of sodium to ensure that the brain is well supplied with blood and is therefore functioning efficiently and transmitting signals effectively. Research also demonstrates that salt may influence the immune system as it influences the function of some of the immune cells which may be involved in inflammation processes [11]. Salt has been seen to influence hormones, especially in the adrenal glands, where it helps in the release of aldosterone which is a hormone that is involved in the absorption of sodium and the excretion of potassium to maintain electrolyte balance. Therefore, salt is essential for hydration, signal conduction along the nerves, muscle activity, pH maintenance, blood pressure, osmotic stability, absorption of nutrients, cognitive functions, modulation of the immune system, and hormone secretion and release, thus maintaining good health [12,13].

## 3. How the Body Normally Regulates High Salt Levels (Figure 2)

The human body has various compensatory mechanisms that help to maintain sodium levels, especially in situations where the body is exposed to high sodium content. All these physiological regulatory mechanisms are important in the maintenance of sodium homeostasis to compensate for and minimize the damage that high levels of sodium can cause to the circulatory and renal systems. These mechanisms are integrated and involve the cardiovascular, renal, hormonal, and nervous systems in the management of high salt intake, as shown in the figure and expounded in the following diagram.

**Figure 2:**
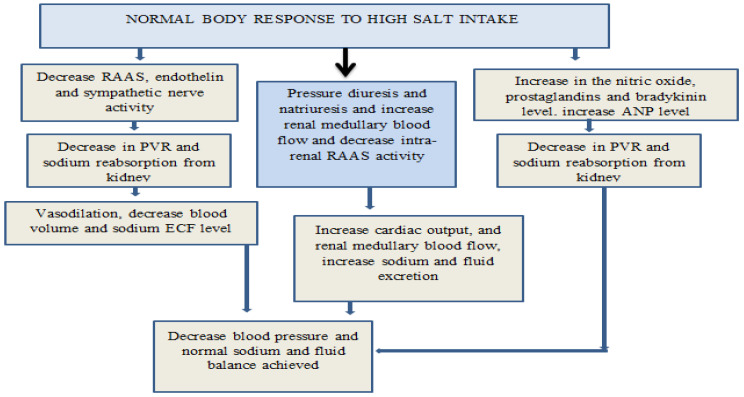
Normal body responses to high salt intake.

## 4. Cardiovascular and Renal Hemodynamic Response to High Salt Intake for Maintaining Homeostasis

### 4.1. Pressure Diuresis and Natriuresis

Under the influence of high salt intake, the body’s response is an increase in the blood volume and a transient rise in the blood pressure to ensure the process of pressure diuresis and natriuresis, and this is the key player for maintaining blood volume, as described by Guyton. High salt intake initially increases plasma volume due to osmotic water retention, leading to raised cardiac output and arterial blood pressure. This rise in the blood volume and blood pressure triggers enhance renal perfusion and interstitial hydrostatic pressure, leading to a rise in the blood flow to the kidneys. The resulting surge in renal flow blood also elevates the glomerular rate (GFR), allowing larger filtration of sodium and water in the renal glomerular filtration bed, thereby preventing chronic sodium and water retention [14]. The reflection of this transient rise in the renal medullary blood flow and renal interstitial pressure, due to medullary vascular resistance, brings a prompt reduction in hyperosmotic renal medullary gradient, preventing tubular sodium and water reabsorption and promoting their excretion [15]. Additionally, sodium transporters NHE3 in the proximal tubules, NCC in the distal convoluted tubules, and ENaC in the collecting ducts are modulated to ensure more sodium and water remain in the filtrate without undergoing tubular reabsorption, thus enabling natriuresis and diuresis. The combined effects of altered tubular sodium transport increased renal interstitial pressure, medullary blood flow, and reduced medullary resistance reinforce the pressure–natriuresis and pressure–diuresis mechanisms representing a coordinated physiological adaptation that prevents excessive salt retention and blood volume expansion and blood pressure, thus preventing the risks of salt-induced hypertension and organ damage and thereby protecting cardiovascular and renal function [16]. The process is driven by the coordinated actions of neural and hormonal pathways, including the renin–angiotensin system, the sympathetic nervous system, and hormones such as nitric oxide, prostaglandins, bradykinin, and endothelins. These mechanisms collectively regulate pressure diuresis and natriuresis, enabling efficient sodium and water excretion while preserving fluid balance and blood pressure [17]. Let us now explore how these pathways interact to facilitate pressure diuresis and natriuresis.

### 4.2. Hormonal Modulation via the RAAS

The renin–angiotensin–aldosterone system (RAAS) plays a vital role in regulating sodium balance by facilitating sodium reabsorption in the kidneys. Under normal conditions, RAAS helps maintain blood pressure by promoting sodium reabsorption and water retention. Angiotensin II, a key component of RAAS, is produced in response to low blood pressure or reduced sodium delivery to the distal nephron [13]. It increases sodium reabsorption in the proximal tubule and distal nephron by stimulating sodium transporters and acts as a potent vasoconstrictor, reducing renal blood flow and glomerular filtration rate (GFR) while raising vascular resistance and blood pressure. While aldosterone increases sodium reabsorption from the distal tubule of the nephron, during high salt intake, RAAS activity is suppressed, leading to reduced secretion of renin, angiotensin II, and aldosterone. Decreased angiotensin also decreases vascular resistance; this suppression decreases sodium reabsorption in the kidneys, facilitating natriuresis and diuresis. High sodium intake inhibits renin release from the juxtaglomerular cells, resulting in lower production of angiotensin II and aldosterone. With reduced aldosterone levels, sodium reabsorption in the distal nephron and collecting ducts (via epithelial sodium channels, ENaC) is further diminished, enhancing sodium excretion and maintaining fluid balance [18].

### 4.3. Sympathetic Nervous System

The sympathetic nervous system releases norepinephrine, which acts on alpha-adrenergic receptors to induce vasoconstriction. This vasoconstriction, particularly in systemic arterioles, increases vascular resistance and raises blood pressure. In the kidneys, norepinephrine released by renal sympathetic nerves constricts renal arterioles, reducing renal blood flow and glomerular filtration rate (GFR). Additionally, norepinephrine stimulates sodium reabsorption in the proximal tubule and the thick ascending limb of the loop of Henle, promoting sodium and water retention [19,20,21].

During high salt intake, sympathetic activity is reduced, decreasing norepinephrine release. This reduction minimizes sodium reabsorption, enhancing natriuresis and diuresis, thereby promoting sodium excretion and lowering blood pressure. Decreased sympathetic nerve activity also prevents excessive systemic and renal vasoconstriction, allowing vasodilation, increased filtration, and supporting pressure natriuresis and diuresis [22,23].

## 5. Baroreceptor-Mediated Regulation of Blood Pressure and Fluid Balance in Response to High Salt Intake

Baroreceptors play a crucial role in detecting changes in blood pressure and facilitating short-term adaptations to high salt intake through pressure diuresis and natriuresis. These mechanoreceptors, located in the aortic arch and carotid sinuses, sense arterial wall stretch caused by elevated blood pressure due to increased blood volume from high salt intake. Upon detecting this rise, baroreceptors transmit signals to the cardiovascular control centers in the medulla, prompting adjustments in autonomic nervous system output to regulate blood pressure. This reflex inhibits sympathetic nervous system (SNS) activity, reducing vasoconstriction and promoting vasodilation [24,25]. The resulting decrease in vascular resistance improves renal perfusion and increases glomerular filtration rate (GFR), facilitating sodium and water excretion. Baroreceptor-mediated reduction in SNS activity also suppresses renin release from juxtaglomerular cells, leading to lower levels of angiotensin II and aldosterone. Together with the reduction in the sodium reabsorption in the renal tubules, these effects are involved in the maintenance of fluid balance and blood pressure. Through stimulating pressure diuresis and natriuresis baroreceptors can therefore control blood pressure and avoid complications of saltwater intoxication [26,27,28].

## 6. Vasodilators in Pressure Diuresis and Natriuresis: Maintaining Fluid and Sodium Balance During High Salt Intake

Vasodilators such as nitric oxide (NO), prostaglandins, atrial natriuretic peptide (ANP), endothelin, and bradykinin play a crucial role in regulating the body’s response to high salt intake. These vasodilators enhance renal blood flow, which improves filtration and facilitates the excretion of sodium and water. Increased medullary blood flow and reduced sodium reabsorption in the nephron decrease the kidneys’ ability to concentrate urine, promoting water loss and contributing to diuresis. These intrarenal adaptations work in harmony to eliminate excess sodium and water, aiding in blood pressure regulation and maintaining fluid balance.

Nitric oxide: Nitric oxide (NO) is an endothelium-derived vasodilator that is synthesized by the endothelial cells lining the blood vessels. There is also a growing understanding that NO is produced constitutively by the endothelium of the kidney and that NO plays an important role in the control of renal circulation and tubular function. Bradykinin and acetylcholine dilate the renal vasculature through the release of NO which results in increased diuresis and natriuresis [29]. Inhibition of basal NO production has been found to lead to a reduction in RBF and sodium transport.NO dilates renal blood vessels and thus increases RBF and GFR which in turn helps in the removal of excess sodium and water from the body in the form of natriuresis and diuresis respectively. NO acts directly on the sodium transporters like NHE3 in the proximal tubule and suppresses the sodium reabsorption, thus preventing salt retention during a high-salt diet. In addition, NO dilates the blood vessels in the renal medulla, thus reducing the medullary osmotic gradient and the urinary concentrating capacity which in turn increases sodium and water excretion. In systemic circulation, NO is synthesized by the endothelial cells because of shear stress and other stimuli and is a potent vasodilator. During a high-salt diet, NO production is upregulated to dilate blood vessels and allow for the expansion of blood volume that occurs due to salt-induced hydration. NO relaxes the blood vessels and thus reduces peripheral vascular resistance and systemic blood pressure, thus counteracting the effects of high salt intake on blood pressure. It also guarantees sufficient blood supply to the vital organs including the kidneys, heart, and brain [30].

## 7. Atrial Natriuretic Factor (ANP)

Atrial natriuretic peptide (ANP) is secreted from the atria of the heart when the volume of blood in the circulation and the pressure increases, which happens when one takes in a lot of salt. ANP relaxes the afferent arterioles and constricts the efferent arterioles in the kidneys thereby increasing the GFR and the excretion of sodium and water. In the collecting ducts, it acts to inhibit the absorption of sodium by decreasing the number of open epithelial sodium channels (ENaC), thus increasing sodium excretion or natriuresis and water through osmosis, which results in increased urine output to decrease blood volume and pressure in the arteries [31]. Besides these renal functions, ANP also causes systemic vasodilation mainly in the arterioles, which results in decreased vascular resistance and thus decrease in blood pressure. Through its function of reducing blood volume and arterial pressure, ANP is crucial in the management of high salt intake and regulation of the cardiovascular system [32].

## 8. Prostaglandins

These prostaglandins, such as PGE2 and prostacyclin (PGI2), are produced by endothelial and smooth muscle cells in the blood vessels which result in vasodilation after relaxing the vascular smooth muscle. This vasodilation results in decreased systemic vascular resistance and therefore assists in the reduction in blood pressure during high sodium intake. Also, prostaglandins stimulate blood supply to organs like the heart and kidneys, thus guaranteeing proper perfusion and assisting the body in adapting to high salt intake. Prostaglandins that are synthesized in the renal cortex and medulla in response to a rise in renal perfusion pressure are known to regulate renal and systemic functions during a high-salt diet. One of the prostaglandins, PGE2, is a vasodilator that enhances the renal blood flow and the GFR. PGE2 also has an inhibitory effect on sodium transport in the thick ascending limb of the loop of Henle and the collecting ducts, thus increasing the sodium excretion. Prostaglandins balance the actions of vasoconstrictors such as angiotensin II to allow for proper renal perfusion as well as sodium excretion during high salt intake [33].

## 9. Bradykinin

Bradykinin is a kinin that is produced through the kallikrein-kinin system and acts on the renal vasculature to dilate the blood vessels and increase renal blood flow and the GFR. It also has the effect of reducing the reabsorption of sodium in the proximal tubule and the distal nephron, thus increasing sodium excretion. Released in the kidneys, as well as in many other tissues, including the blood vessels, bradykinin is a potent vasodilator that stimulates the production of NO and PGI2 by endothelial cells. This vasodilation is beneficial in reducing the effects of high salt intake on blood pressure and maintaining fluid volume [34].

Endothelin: Endothelin, an endothelium-derived peptide, is the most potent vasoconstrictor that is produced by endothelial cells and is involved in the control of vascular resistance and blood pressure. It causes contraction that is very powerful and long-lasting and thus increases vascular resistance and blood pressure. In the kidneys, endothelin acts on the renal blood vessels to constrict them thus reducing the renal blood flow and the GFR especially when the salt intake is low. In states of high salt intake, endothelin tone is reduced to allow for sodium excretion and to regulate fluid volume. This reduction affects the sodium transport in the collecting ducts of the kidney to cause natriuresis. The reduced endothelin activity also favors vasodilation which increases the renal blood flow and sodium excretion. Also, the vasoconstrictive effects of endothelin are antagonized by vasodilators such as nitric oxide and prostaglandins when the salt intake is high to maintain normal blood pressure and tissue perfusion [35].

Thus, collective interaction between the renal and cardiovascular systems under the influence of various hormonal and neural mechanisms ensures effective regulation of blood pressure and fluid balance in response to high salt intake. These processes safeguard the body by promoting the excretion of excess sodium and water, preventing prolonged elevation of blood pressure, and protecting vital organs such as the kidneys, heart, and blood vessels. This coordinated response highlights the body’s remarkable ability to adapt to dietary salt changes and maintain homeostasis, mitigating the harmful effects of salt-induced pressure and volume overload. Hence, it is understood from the above information that, in response to a high salt load, arterial pressure increases, enhancing renal perfusion, GFR, and renal medullary blood flow, while reducing sodium reabsorption. As renal perfusion pressure increases, renal interstitial hydrostatic pressure (RIHP) rises, triggering natriuresis through the action of renal autacoids such as angiotensin II, nitric oxide (NO), prostaglandins, and kinins. Angiotensin II, a key sodium-retaining hormone, influences medullary flow, RIHP, and pressure natriuresis (PN). Increased perfusion pressure suppresses intrarenal angiotensin II levels, reducing sodium reabsorption and medullary vascular resistance [36]. The rise in RIHP, independent of overall renal blood flow, likely results from increased medullary perfusion and reduced medullary vascular resistance. RIHP also modulates sodium reabsorption by altering tight junction permeability in proximal tubules, redistributing sodium transporters, and promoting the release of autacoids like prostaglandin E2. Without these regulatory processes, excessive salt consumption can lead to elevated blood pressure and localized organ damage, including harm to the kidneys, heart, and blood vessels. This coordinated response helps restore normal blood pressure and maintain fluid homeostasis. The integration of hemodynamic, hormonal, and neural mechanisms ensures that blood pressure does not remain elevated for long after a high salt intake, preventing damage to the cardiovascular and renal systems.

## 10. The Lifelong Effects of Excessive Sodium: From Early Childhood Exposure to Adult Health Challenges

The body possesses robust mechanisms to regulate sodium and maintain fluid balance, but persistent high sodium levels can overwhelm these systems, leading to detrimental adaptations. Initially, the body compensates by enhancing sodium excretion through hormonal and neural pathways. However, with prolonged high salt consumption, these regulatory systems may reset, resulting in higher baseline blood pressure. This resetting involves various physiological changes, including baroreceptor adaptation, renal processes to excrete sodium and water, and alterations in vascular, neural, and hormonal functions, collectively contributing to the onset and persistence of hypertension. If this salt-induced elevation in blood pressure becomes chronic, it can increase the risk of hypertension, stroke, kidney damage, and cardiovascular disease.

If we consider the present scenario with the rampant prevalence of hypertension among adults and teens, the impact of high sodium intake begins early in life, as children are frequently exposed to diets rich in salt and sugar, particularly through fast food and processed products. This trend is exacerbated by reduced physical activity and increased screen time, contributing to calorie intake without adequate calorie expenditure. The cumulative effects of excessive sodium consumption increase the risk of developing hypertension and related conditions. Studies have shown that early exposure to high salt intake can lead to the adaptation of sodium and fluid regulatory mechanisms, leading to setting a high baseline blood pressure level, predisposing individuals to risk of cardiovascular and renal diseases, and predisposing one to stroke and other high-salt-induced organ damage later in life. Cardiovascular diseases occur in adulthood, but the underlying vascular alterations begin very early in childhood and are related to the presence of risk factors such as arterial hypertension [37]. The prevalence of essential arterial hypertension in children and adolescents has grown considerably in the last few decades, making this disease a major clinical problem in the pediatric age. The pathogenesis of arterial hypertension is multifactorial, with one of the components being represented by incorrect eating habits. The current dietary intake of salt in many children surpasses recommended levels by health organizations, laying the foundation for health problems.

The ancestral diets did not contain much salt and sugar, while the modern diets have changed a lot. Currently, adults consume about twice the amount of salt that the World Health Organization recommends. Hypertension has many causes and is influenced by genetics, environment, and lifestyle, and salt intake has been proven to have a lot to do with it. Some works have analyzed the consequences of consuming salt over a long period of time. For instance, Hoffman’s study revealed that infants who were given normal levels of sodium in their diet for twenty-five weeks had higher systolic blood pressure as compared with the infants who were on a low-sodium diet. When the same subjects were tracked down after 15 years, it was discovered that a low sodium intake during infancy resulted in a decrease in blood pressure of 3.6 mmHg. This shows the need to take salt intake to the minimum right from infancy to avoid hypertension and other related diseases [37].

The consequences of high salt intake during childhood, such as obesity, hypertension, and increased salt sensitivity, can persist in adulthood, contributing to higher mortality and morbidity. Early childhood is critical for overall health and well-being and is an ideal time for preventive interventions. The first 1000 days of life are particularly significant for brain and body development, during which excessive salt intake can be harmful. The major sensitive period for neurological development is the first 1000 days. Some of the key primary brain structures and functions that develop include the sensory system, hippocampus, myelination, and the monoamine neurotransmitter systems. These are most crucial for brain and body growth, for language and sensory system development of hearing and vision as well as for higher cognitive functions. It is a period of very fast neurodevelopment and is more influenced by their environment [38,39]. Foods like table salt, processed meats, bread, snacks, and condiments are common sources of high sodium. Studies have highlighted that many children consume excessive amounts of salt, which can have lasting health effects. The WHO recommends that children aged 24 months to 15 years should have adjusted sodium intake levels in line with their energy requirements to maintain health and reduce the risk of hypertension and other related diseases later in life. Encouraging balanced eating habits with appropriate sodium intake is crucial to support long-term health and prevent chronic illnesses. Additionally, Salt sensitivity plays a critical role in hypertension, characterized by individual variations in blood pressure response to dietary salt. About 50–60% of hypertensive patients are salt-sensitive, influenced by impaired renal function, reduced pressure natriuresis, overactive RAAS, and increased sympathetic activity. Genetic polymorphisms affecting sodium handling contribute to this sensitivity, often appearing early in life [40,41]. The term salt sensitivity of blood pressure is defined as a physiological trait whereby the blood pressure of some members of the population varies with salt intake. BP response to a change in salt intake is not uniform. The kidneys become unable to excrete a sufficient amount of sodium in response to a high sodium intake. About 50–60% of hypertensive subjects are salt-sensitive. Salt sensitivity is more prevalent in elderly people, females, obese subjects, and patients with chronic kidney disease. Salt sensitivity seems to be influenced by genes, race/ethnicity, age, sex, BMI, and diet [10,40].

High-sodium diets during childhood can enhance salt sensitivity, increasing the risk of early-onset hypertension and long-term cardiovascular and renal issues. Early identification and reducing sodium intake can mitigate these risks, as children’s bodies are more responsive to dietary changes. Personalized interventions based on genetic predispositions can prevent or manage salt sensitivity, promoting lifelong cardiovascular health.

## 11. The Body’s Adaptation to Chronic High Sodium Intake from Sodium Balance to Hypertension

Chronic high salt intake prompts the body to adapt its normal salt-regulating mechanisms, often leading to elevated blood pressure over time. Initially, the body responds to high salt intake by increasing sodium and water excretion through normal regulatory mechanisms. However, with sustained high sodium consumption, these regulatory processes can become overwhelmed or reset to maintain higher baseline blood pressure. Key adaptations include changes in renal function, altered pressure–natriuresis response, decreased suppression of the renin–angiotensin–aldosterone system (RAAS), increased sympathetic nervous system activity, and hormonal imbalance. These adaptations collectively reduce the efficiency of sodium excretion and promote fluid retention, contributing to chronic hypertension.

## 12. Impaired Pressure–Natriuresis Response (Figure 3)

Pressure diuresis and natriuresis represent the body’s primary mechanisms for managing high salt intake by excreting excess sodium and osmotically induced fluid volume. These processes involve intricate interactions between the kidneys, cardiovascular system, and various hormonal and neural pathways. However, prolonged high salt intake triggers adaptive changes in these regulatory mechanisms, reducing the efficacy of pressure diuresis and natriuresis. Consequently, the mechanisms that initially enhance sodium excretion in response to a slight increase in blood pressure require a higher baseline pressure to achieve sodium excretion and the kidneys require a higher blood pressure to trigger the same level of sodium excretion, leading to a reset in blood pressure regulation. This adaptation often results in a new, elevated baseline for blood pressure. Over time, this diminished response impairs the body’s ability to excrete sodium effectively, leading to increased blood pressure and a heightened risk of hypertension. Let us explore the various mechanisms that adapt to chronic high salt intake and alter the regulation of pressure diuresis and natriuresis. Chronic high salt intake alters the pressure–natriuresis curve by diminishing the kidneys’ sensitivity to pressure changes, requiring higher arterial pressure to excrete sodium effectively [36]. This adaptation impairs natriuretic response due to reduced renal perfusion, heightened renal sympathetic nerve activity, and increased activity of the renin–angiotensin–aldosterone system (RAAS). Additionally, prolonged salt consumption promotes sodium retention through enhanced reabsorption in the nephron, maintaining elevated blood volume and exacerbating hypertension. Vascular and hormonal changes, including reduced nitric oxide production, increased oxidative stress, and vascular stiffness, further impair renal perfusion and sodium excretion, collectively contributing to the development and progression of hypertension. Pressure diuresis and natriuresis are key physiological processes activated in response to increased arterial pressure, involving the kidneys, cardiovascular system, hormonal pathways, neural mechanisms, and baroreceptor signaling. These processes work together to promote sodium (natriuresis) and water (diuresis) excretion, maintaining blood pressure and fluid homeostasis.

**Figure 3:**
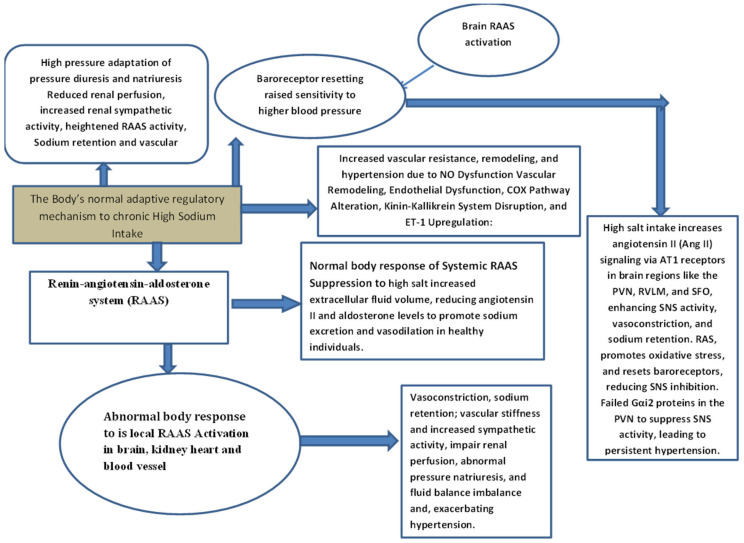
The body’s adaptation to chronic high sodium intake from sodium balance to hypertension.

## 13. Adaptation and Resetting of Baroreceptors

Baroreceptors are essential for short-term blood pressure regulation, detecting even minor increases in baseline pressure. However, chronic exposure to high salt intake and the consequent sustained rise in blood pressure can lead to adaptive changes in their function. This adaptation involves resetting their sensitivity to higher baseline pressure, which in turn shifts the pressure diuresis and natriuresis mechanisms to operate at elevated pressure levels. Baroreceptors initially respond to changes in blood pressure by sending feedback to the central nervous system to modulate autonomic nervous system output and maintain blood pressure within normal ranges. However, with chronic high salt intake, which leads to a sustained increase in blood volume and arterial pressure, baroreceptors adapt by resetting their threshold of sensitivity to higher pressure levels. This resetting means that what was previously perceived as high pressure becomes the new “normal”, leading to a reduced response to elevated blood pressure. The adaptation of baroreceptors involves changes in the neural activity and mechanical properties of the arterial wall where baroreceptors are located. Over time, these sensors become less responsive to pressure changes that would normally trigger regulatory responses [42]. The baroreceptors’ firing rate decreases in response to sustained high pressure, signaling less frequently to the cardiovascular centers in the brain. The set point for baroreceptor activation is reset to higher pressure levels, reducing the reflexive response to control blood pressure. When baroreceptors reset to a higher pressure level, their ability to trigger responses that support pressure diuresis and natriuresis becomes impaired [43]. Normally, baroreceptors help reduce sympathetic nervous system (SNS) activity when blood pressure is high, promoting vasodilation and increased renal perfusion. However, with a reset threshold, baroreceptors do not respond as strongly to moderate increases in pressure, leading to sustained SNS activity, continued vasoconstriction, and reduced renal blood flow. The baroreceptor-mediated feedback loop that helps suppress the renin release is also affected. With a higher set point, the reflex to decrease renin and subsequent angiotensin II and aldosterone levels are weakened. This leads to sustained sodium and water retention rather than excretion, undermining the pressure–natriuresis response [44]. The usual increase in glomerular filtration rate (GFR) that supports pressure diuresis may not be as effectively triggered, as the baroreceptors do not signal the need for reduced SNS output and vasodilation. This limits the kidneys’ ability to excrete sodium and water efficiently. Diminished baroreceptor response leads to continued SNS activity, impaired suppression of RAAS, and reduced GFR modulation, all of which contribute to sodium and fluid retention and sustained high blood pressure [44]. The resetting of baroreceptor sensitivity to a higher blood pressure level allows the body to adapt to chronic high salt intake by accepting an elevated baseline pressure as normal. This adaptation reduces the baroreceptors’ ability to initiate the necessary autonomic and hormonal changes that promote pressure diuresis and natriuresis [45]. As a result, blood pressure remains elevated, contributing to chronic hypertension. In understanding the mechanism behind baroreflex adaptation, it is suggested that high salt intake impairs the baroreflex, contributing to hypertension through multiple interconnected mechanisms. Oxidative stress, neuroinflammation, hormonal dysregulation, and neurotransmitter imbalances collectively contribute to the central resetting of baroreflex pathways under high salt intake. Elevated salt levels increase reactive oxygen species (ROS) production in the central nervous system, particularly in the paraventricular nucleus (PVN). This oxidative stress enhances sympathetic nervous system activity and shifts the baroreflex set point to a higher blood pressure range. High salt triggers neuroinflammation with microglial activation and upregulation of proinflammatory cytokines such as TNFα in areas including the PVN, NTS, and basolateral amygdala. This affects neuronal function and decreases baroreflex sensitivity. High salt affects the renin–angiotensin–aldosterone system, thus increasing the levels of angiotensin II and aldosterone in the central nervous system pathways. These hormones increase the sympathetic tone and are also involved in the setting of baroreflex sensitivity [46]. Changes in glutamate and GABA ratios in the PVN and RVLM during salt intake affect the baroreflex control, namely a reduction in inhibitory GABAergic signaling and an enhancement of the excitatory drive and sympathetic outflow [47]. Recent research showed that testosterone aggravated the adverse impact of high salt on baroreflex function in male Sprague Dawley rats. Castration reversed the effect of salt on baroreflex. These findings show that oxidative stress, inflammation, hormonal mechanisms, and central neurotransmitter systems are involved in the baroreflex resetting in high salt intake [48,49].

## 14. Impact of High Salt Intake on RAAS Regulation and Hypertension

Chronic high salt intake has a profound impact on blood pressure, affecting both cardiovascular and renal health. The renin–angiotensin–aldosterone system (RAAS) is critical in regulating blood pressure by maintaining fluid and sodium balance. However, prolonged high salt consumption can lead to maladaptations or dysfunction in these mechanisms, increasing susceptibility to salt-induced hypertension and organ damage. The RAAS functions not only systemically but also locally in organs such as the brain, kidneys, blood vessels, and heart tissue. Disruption of both systemic and local RAAS due to high salt intake contributes to elevated blood pressure and damage to these organs.

## 15. Impact of Chronic High Salt Intake on the Renin–Angiotensin–Aldosterone System (RAAS) and Related Organ Damage

The renin–angiotensin–aldosterone system (RAAS) is a critical regulator of sodium and fluid balance, blood pressure, and vascular tone. It acts both systemically and locally in organs like the kidneys, heart, and blood vessels. While RAAS typically maintains homeostasis, chronic high salt intake can alter its function in ways that promote hypertension. High salt intake disrupts the balance of the renin–angiotensin–aldosterone system (RAAS), which regulates sodium balance and blood pressure.

In salt-sensitive individuals, RAAS dysregulation can lead to inappropriate angiotensin II and aldosterone activity, promoting fluid retention, vascular stiffness, and sympathetic overdrive. These changes contribute to sustained hypertension, impaired blood pressure regulation, and increased risks of kidney and cardiovascular damage.

## 16. Systemic RAAS Suppression

Salt intake is also known to inhibit the secretion of renin from the juxtaglomerular apparatus of the kidney due to feedback inhibition by increased extracellular fluid volume. This reduces the secretion of systemic Ang II and aldosterone, which in turn increases sodium excretion and dilation of blood vessels in normal subjects. (RAAS), typically suppressing renin release due to increased renal perfusion and sodium delivery to the macula densa [50]. This suppression reduces angiotensin II and aldosterone levels, facilitating sodium and water excretion to maintain fluid balance. However, in salt-sensitive individuals, RAAS dysregulation paradoxically sustains vasoconstriction, sodium retention, and hypertension. These individuals often exhibit heightened sensitivity to angiotensin II, while aldosterone escape allows continued sodium reabsorption despite volume expansion [50]. Interactions between systemic RAAS suppression and local RAAS activation in organs like the kidneys and heart amplify hypertensive effects. High salt intake induced angiotensin vascular stiffness, further promoting a pro-hypertensive state. Additionally, high salt intake enhances sympathetic nervous system activity through central RAAS pathways, increasing vasoconstriction and reducing renal perfusion, impairing pressure natriuresis and exacerbating fluid retention and hypertension [10,40].

## 17. Local RAAS Activation

Tissue-Specific Effects: Despite systemic suppression, local RAAS in organs such as the brain, kidneys, blood vessels, and heart remain activated under chronic high salt intake. While systemic RAAS activity is typically suppressed due to increased renal perfusion and sodium delivery, local RAAS activation in the kidneys, blood vessels, and central nervous system persists, driving hypertensive effects. This local activation enhances sodium retention, vasoconstriction, and oxidative stress while impairing pressure natriuresis. Systemic suppression prevents fluid accumulation but does not reverse the process of local RAAS activation, thus worsening hypertension and organ dysfunction [32].

Brain RAAS Activation: The brain RAAS is a critical regulator of the sympathetic nervous system (SNS) and blood pressure. It involves local production of angiotensinogen, renin, ACE, and Ang II within the brain, particularly in regions like the hypothalamus and subfornical organ (SFO) [51]. Ang II binds to AT1 receptors in key areas, increasing sympathetic outflow and raising blood pressure through vasoconstriction and increased cardiac output. Chronic high levels of Ang II in the brain reset baroreceptor sensitivity, desensitizing the baroreflex to high blood pressure and reinforcing a higher blood pressure set point. Ang II also induces ROS production; these mechanisms contribute to sustained hypertension [52,53].

Kidney RAAS Dysregulation: Sustained intrarenal Ang II promotes sodium reabsorption via Na^+^/H^+^ exchangers and Na^+^/K^+^ ATPase in proximal tubules, leading to sodium retention, glomerular hyperfiltration, and chronic kidney damage. The intrarenal RAAS plays a pivotal role in sodium balance and blood pressure regulation. In salt-sensitive individuals or under chronic high salt conditions, local oxidative stress and inflammatory mediators upregulate angiotensinogen and ACE expression in renal tissue. This leads to sodium retention, efferent arteriole constriction, glomerular hyperfiltration, and chronic kidney damage. This sustains intrarenal Ang II production and enhances sodium reabsorption via the Na^+^/H^+^ exchanger and Na^+^/K^+^ ATPase in the proximal tubules. The persistent activity of intrarenal Ang II promotes sodium retention and volume expansion, leading to increased blood pressure [54,55]. Persistent intrarenal Ang II is associated with a blunted pressure–natriuresis response, where higher blood pressure is required to excrete the same amount of sodium. The blood-pressure-independent effect of high salt results in albuminuria, oxidative stress, severe renal arteriolar damage, interstitial fibrosis, increased glomerular filtration hydrostatic pressure, and end-stage renal disease. A high-salt diet increases blood volume and pressure, which can damage the glomerular membrane and impair glomerular filtration. High salt intake also causes inflammation and fibrosis in renal tubules, reducing their ability to reabsorb essential substances and excrete waste, leading to chronic kidney disease. Additionally, high salt intake induces the production of reactive oxygen species and inflammatory cytokines, causing oxidative stress and further renal damage [56,57].

Cardiac and Vascular Effects: In the heart and vasculature, Angiotensin II (Ang II) plays a central role in promoting fibrosis, vascular remodeling, and hypertrophy, which reduce elasticity and contribute to sustained high blood pressure. Chronic high salt intake exacerbates local Ang II activity in the heart by activating AT1 receptors, triggering signaling pathways such as MAPK and JAK/STAT. These pathways drive cardiac hypertrophy and fibrosis by activating fibroblast proliferation and collagen synthesis, thereby increasing the stiffness and size of the heart. In addition, Ang II enhances the production of reactive oxygen species in the cardiac tissue through the activation of NADPH oxidase, thus leading to oxidative stress that affects the cardiac cells and causes hypertrophy and heart failure. Oxidative stress, associated with high salt intake, increases ROS production and decreases NO bioavailability [58,59,60,61,62]. This results in endothelial dysfunction, vasoconstriction, and vascular stiffness, which worsen the cardiovascular condition. It also increases the levels of proinflammatory cytokines such as IL-6 and TNF-α, which cause chronic inflammation, fibrosis, and immune cell activation. These processes enhance organ dysfunction mainly in the heart and kidneys, while redox-sensitive transcription factors including NF-κB get activated to express pro-inflammatory cytokines and adhesion molecules. These molecules solicit immune cells thereby promoting chronic inflammation, vascular cell growth, and remodeling. Also, the increased activity of matrix metalloproteinases (MMPs) is involved in the thickening of the vessel walls and a reduction in their elasticity, which in turn worsens the vascular function [61]. High salt intake over a long period causes dysregulation of the renin–angiotensin–aldosterone system, where there is suppression of the system, but local enhancement of the system leads to high blood pressure and organ damage. New knowledge on tissue-dependent RAAS mechanisms and therapeutic strategies helps in preventing salt-induced diseases and their complications for the benefit of cardiovascular and renal systems [62,63,64,65,66].

## 18. Sympathetic Nervous System (SNS) Overactivity

High sodium intake results in sustained stimulation of the sympathetic nervous system (SNS), which leads to increased heart rate and vasoconstriction and thus maintains high blood pressure. When large amounts of salt are consumed over a long period of time, it leads to SNS overactivity, where the activity of the SNS is constantly high. This means that the blood vessels remain constricted, the heart rate is elevated, and the blood pressure is set at a higher level. At first, baroreceptor feedback feels high pressure in the blood and reduces SNS activity [67].

However, with persistent high salt intake, baroreceptors reset to a higher pressure threshold, reducing their inhibitory effect on the SNS. This allows continued SNS activation, promoting vasoconstriction, sodium retention, and blunting of the pressure–natriuresis response, further sustaining elevated blood pressure. High salt intake also triggers excessive norepinephrine release, increasing heart rate and vasoconstriction, while contributing to renal vasoconstriction, reduced renal perfusion, and enhanced sodium reabsorption. These effects aggravate volume-dependent hypertension and place a strain on cardiovascular and renal function [68,69,70]. Both experimental and clinical studies show that high salt diets elevate plasma and cerebrospinal fluid (CSF) sodium levels, stimulating sodium-sensitive receptors and osmoreceptors that increase SNS activity and arterial blood pressure, particularly in salt-sensitive individuals. Central sympathetic networks are also modulated by chronic high salt intake, becoming hyperresponsive to neurotransmitters and reflex stimuli [71]. Regions such as the rostral ventrolateral medulla (RVLM) and the forebrain lamina terminalis, including the organum vasculosum of the lamina terminalis (OVLT), subfornical organ (SFO), and median preoptic nucleus (MnPO), play a pivotal role in this process. These areas, which lack a complete blood-brain barrier, respond to elevated circulating sodium levels by increasing neuronal activation. Markers like Fos are upregulated in the OVLT and SFO, while lesions in these regions attenuate hypertensive responses, underscoring the importance of osmosensitive pathways in the hypertensive effects of chronic salt consumption. High salt intake also disrupts the renin–angiotensin system (RAS), leading to increased production of angiotensin II (Ang II), which enhances SNS activity [72,73]. Ang II binds to receptors in the brain and stimulates the sympathetic nervous system, thus contributing to the increase in blood pressure. Several factors including mineralocorticoid receptors, aldosterone, corticone, epithelial sodium channels, and Ang II are involved in the activation of the SNS, and oxidative stress is considered as one of the most important factors. The current studies show that Gαi2-protein-mediated signaling in the hypothalamic paraventricular nucleus (PVN) is a protective mechanism against salt-induced hypertension. In salt-tolerant subjects, high salt dieting increases Gαi2 proteins in the PVN of the brain, which leads to sympathoinhibition and thus helps in maintaining sodium homeostasis without developing hypertension. But in salt-sensitive subjects, these mechanisms are not followed due to the failure of the PVN to upregulate Gαi2 proteins, thus resulting in salt-sensitive hypertension. These findings have highlighted the importance of the central SNS pathways, oxidative stress, and the Gαi2 protein in the pathophysiology of salt-sensitive hypertension [74]. Such targets may, therefore, provide new therapeutic approaches to antagonize the effects of prolonged high salt intake and enhance the management of hypertension and other cardiovascular and renal diseases in the long term.

## 19. Vasodilatory Factors

High salt intake causes molecular alterations that affect vasodilation and enhance local Ang II production in different tissues. The generation of ROS, endothelial dysfunction, and the activation of pro-inflammatory and profibrotic pathways are also involved in these processes. These mechanisms all work in concert to increase sodium reabsorption, vascular tone, and tissue growth and ultimately lead to the development of hypertension over a longer period which underlines the importance of salt intake control and the measures that must be taken in order to maintain the health of the cardiovascular and renal systems.

## 20. Impact of High Salt Intake on Nitric Oxide (NO) and Vascular Remodeling

Salt is known to have severe impacts on the vascular system, mainly because it interferes with nitric oxide production and promotes vascular remodeling through oxidative stress, inflammation, and fibrosis. These processes are thought to contribute to increased stiffness, resistance, and hypertension of the vessels and increase the risk of cardiovascular diseases.

## 21. Nitric Oxide (NO) Dysfunction

High salt intake results in the suppression of endothelial nitric oxide synthase (eNOS), an enzyme that is crucial in the production of NO. This happens in a manner that involves oxidative stress pathways where NADPH oxidase is activated to produce reactive oxygen species (ROS) including superoxide (O2-). ROS react with NO to form (ONOO peroxynitrite), this is a reactive species that consumes NO. This reduces NO levels, thus affecting the endothelium’s function in the regulation of vascular tone and hence increases vascular resistance and blood pressure.

Suckling et al. [75] have shown that high sodium levels are able to transiently stiffen endothelial cells thereby reducing their ability to produce eNOS. This in turn leads to microvascular remodeling, endothelial inflammation as well as functional dysregulation. Extended high salt intake worsens these impacts, as ROS suppresses antioxidant defenses such as superoxide dismutase, hence continuing oxidative injury and reducing NO bioavailability. ROS do not only inhibit NO synthesis but also enhance SNS activity, thus contributing to the development of hypertension. The loss of NO’s basal inhibition of the SNS thus sustains a vicious cycle that enhances the increase in blood pressure and cardiovascular disease [76].

Some groups, such as Black people and postmenopausal women, are at high risk. The increased ADMA, an endogenous inhibitor of eNOS, enhances the reduction in NO bioavailability, thus compromising vasodilation and the ability of the vessels to regulate blood pressure in response to high salt intake. In premenopausal women, estrogen has a protective role through the enhancement of NO production as well as the suppression of Ang II in the renin–angiotensin–aldosterone system. However, estrogen levels decline after menopause, thus disturbing this equilibrium and therefore increasing salt sensitivity, oxidative stress, and the risk of developing hypertension [77]. Surgical menopause trials have shown that salt sensitivity is also twice as high as it was before surgery because of the inability of the body to adequately control the RAAS and NO systems, thus underlining the endocrine system’s importance in the maintenance of vascular function [78].

## 22. Molecular Mechanisms of Vascular Remodeling

Besides NO dysfunction, high salt intake is also involved in the regulation of vascular remodeling through oxidative stress, inflammation, and fibrosis [79]. Superoxide anions formed by NADPH oxidase reduce NO bioavailability and cause endothelial dysfunction and vascular injury [80]. High local concentrations of Ang II that are not suppressed by systemic RAAS also enhance ROS formation and activate redox-sensitive transcription factors including NF-κB and AP-1 [81]. These factors induce the production of pro-inflammatory cytokines, for example, TNF-α and IL-6 that attract immune cells, cause chronic inflammation, and activate endothelium [75,82].

High salt and Ang II also stimulate TGF-β, which leads to the synthesis of ECM proteins through Smad signaling. This leads to remodeling and stiffening of the vessel wall [83]. In the presence of Ang II and other stimuli such as MAPK and JAK/STAT, vascular smooth muscle cells (VSMCs) change from a contractile to a synthetic phenotype. This transformation leads to VSMC proliferation, migration, and synthesis of ECM, which leads to vascular narrowing and increased stiffness. Endothelin-1(ET-1), which is also enhanced during high salt intake, also potentiates vasoconstriction and fibrosis through the ET A receptor. These structural changes are compounded by weakened antioxidant defenses, creating a vicious cycle of oxidative stress, inflammation, and vascular remodeling. Persistent activation of pro-inflammatory and pro-fibrotic pathways sustains vascular damage and hypertension. Thus, high salt intake affects the vascular system and causes the deterioration of NO production, oxidative stress and eNOS dysfunction, vascular remodeling by inflammation, fibrosis, and oxidative damage. These processes are very harmful in salt-sensitive populations and high-risk groups, including Black people and postmenopausal women [84,85,86,87,88,89,90,91,92,93,94,95].

## 23. Endothelial Dysfunction

ROS directly affects endothelial cells and reduces their capacity to synthesize NO. This process is compounded by the stimulation of pro-inflammatory pathways including the NF-κB signaling pathway which is stimulated by high salt intake. The result is a reduction in NO output and compromised vasodilation leading to increased vascular resistance and blood pressure. High salt intake can also affect the intracellular calcium signaling in endothelial cells which is very important for the eNOS function. Dysregulation of calcium processing also affects NO production in the tissue [96,97]. NO is made by endothelial nitric oxide synthase (eNOS) during which L-arginine is converted in the endothelium. It has been demonstrated that a high-salt diet leads to suppression of eNOS and thus less NO is produced. Oxidative stress has been identified to be one of the major factors in this regulation. Superoxide radicals formed because of prolonged high salt intake combined with NO to form peroxynitrate (ONOO^−^), a highly reactive species that quenches NO and affects the endothelial cells [98,99].

Prostaglandins Cyclooxygenase (COX) Pathway: Prostaglandins are synthesized from arachidonic acid via the COX enzymes (COX-1 and COX-2). High salt intake alters the expression of COX-2 in endothelial renal cells, leading to reduced production of prostaglandins such as PGE2 and PGI2, which are critical for vasodilation [100,101,102]. High salt intake enhances oxidative stress, which affects the COX pathway. The presence of ROS disrupts the synthesis of vasodilatory prostaglandins, shifting the balance toward vasoconstrictive prostanoids like thromboxane A2, contributing to increased vascular resistance and reduced blood flow [103,104].

Bradykinin Kinin–Kallikrein System: Bradykinin is produced from kininogen by the action of kallikrein. It binds to B2 receptors on endothelial cells, stimulating the release of NO and prostaglandins. High salt intake reduces the activity of the kinin–kallikrein system by affecting kallikrein synthesis and promoting a pro-inflammatory state. ROS generated by high salt can disrupt bradykinin signaling, reducing its effectiveness in stimulating vasodilation. High salt-induced endothelial dysfunction reduces the number of functional B2 receptors, impairing bradykinin’s ability to activate NO and prostaglandin pathways, leading to reduced vasodilation [105,106,107,108,109].

Endothelin (ET-1) ET-1 Synthesis and Salt Impact: Endothelin-1 is produced by endothelial cells and acts primarily as a vasoconstrictor. Chronic high salt intake upregulates ET-1 gene expression through the activation of salt-sensitive transcription factors like AP-1 and NF-κB. This increase in ET-1 production promotes vasoconstriction and opposes the actions of NO and prostaglandins. ROS not only reduces NO availability but also enhances ET-1 signaling pathways by activating ET_A and ET_B receptors. This leads to sustained vasoconstriction, increased vascular resistance, and promotion of vascular remodeling [110,111,112,113,114,115,116,117,118].

## 24. Non-Osmotic Sodium Storage and Hypertension: The Emerging Roles of Skin as a Key Regulator of Sodium Balance and Blood Pressure

Non-osmotic sodium storage in tissues like skin and epithelial layers is crucial in the regulation of blood pressure and the skin is a reservoir for sodium (Na^+^) accumulation especially in various cardiovascular diseases and risk factors. Even though the precise mechanisms of salt sensitivity and hypertension are not fully understood, renal sodium handling has been considered the primary regulatory factor up to this moment. Nonetheless, new studies are showing that there is a change in the paradigm, and the skin may be the third compartment of sodium homeostasis regulation. Increasing evidence suggests that cutaneous blood flow, salt metabolism, and water balance regulate systemic blood pressure (BP) [119]. The concept of skin sodium storage opens the possibility of an extra buffering system that operates in response to salt intake and BP changes. High levels of sodium in the skin in the long term have been linked to high blood pressure and salt-sensitive hypertension. Research suggests that VEGF-C-mediated lymphangiogenesis plays a critical role in maintaining sodium balance and regulating hypertension. High sodium levels are toxic to the endothelial glycocalyx, which in turn inhibits VEGF-C activation and lymphangiogenesis. This defect leads to a reduction in sodium exit from the skin to the systemic circulation and thus attenuates the vasodilatory response to salt intake. Nevertheless, the recognition of skin—the largest organ in the human body—as a key player in sodium homeostasis opens new possibilities for the pathogenesis of hypertension forecasts for future treatment strategies [120].

Conclusion: The body normally eliminates excess sodium through hormonal and neural regulatory mechanisms involving the cardiovascular and renal systems. However, chronic high salt intake can cause these regulatory systems to adapt to the increased sodium load, leading to sodium accumulation in the body. This results in hypertension and organ damage, including cardiac hypertrophy, renal disease, and vascular stiffness, which further exacerbate hypertension and complications such as stroke, renal failure, and cardiovascular disease. Meanwhile, skin is gaining recognition as a third compartment for sodium storage, playing a crucial role in sodium homeostasis and hypertension. These effects are more pronounced in salt-sensitive individuals. The modern diet, high in salt due to fast foods and processed items, overwhelms these regulatory mechanisms, forcing the body to adapt to persistently elevated sodium levels, increasing the risk of associated health complications.
